# Thinking Inside the Box: A Novel Approach to Smoke Taint Mitigation Trials

**DOI:** 10.3390/molecules27051667

**Published:** 2022-03-03

**Authors:** Colleen Szeto, Renata Ristic, Kerry Wilkinson

**Affiliations:** 1 Department of Wine Science, Waite Research Institute, The University of Adelaide, PMB 1, Glen Osmond, SA 5064, Australia; colleen.szeto@adelaide.edu.au (C.S.); renata.ristic@adelaide.edu.au (R.R.); 2 The Australian Research Council Training Centre for Innovative Wine Production, PMB 1, Glen Osmond, SA 5064, Australia

**Keywords:** activated carbon fabric, anti-transpirant, bushfires, grapes, guaiacol, kaolin, volatile phenols, volatile phenol glycoconjugates, wine

## Abstract

When bushfires occur near wine regions, grapevine exposure to smoke can taint grapes due to the uptake of smoke-derived volatile compounds that can subsequently impart unpleasant smoky, medicinal, burnt rubber and ashy characters to wine. Whereas early research sought to understand the effects of smoke on grapevine physiology, and grape and wine chemistry, research efforts have shifted towards the strategic imperative for effective mitigation strategies. This study evaluated the extent to which excised grape bunches could be reproducibly tainted during smoke exposure in a purpose-built ‘smoke box’. The volatile phenol composition of grapes exposed to smoke for 30 min was similar to that of smoke-affected grapes from field trials involving grapevine exposure to smoke. Some variation was observed between replicate smoke treatments, but implementing appropriate controls and experimental replication enabled the smoke box to be used to successfully evaluate the efficacy of several agrochemical sprays and protective coverings as methods for mitigating the smoke exposure of grapes. Whereas the agrochemical sprays did not provide effective protection from smoke, enclosing grape bunches in activated carbon fabric prevented the uptake of up to 98% of the smoke-derived volatile phenols observed in smoke-affected grapes. As such, the study demonstrated not only a convenient, efficient approach to smoke taint research that overcomes the constraints associated with vineyard-based field trials, but also a promising new strategy for preventing smoke taint.

## 1. Introduction

Grape growers and winemakers are keenly aware of the impacts of climate change on grape production [1] and have already begun adapting viticultural practices in response to warmer and drier growing conditions, for example, through the use of heat- and drought-tolerant cultivars and rootstocks [2], in-canopy sprinkler systems to mitigate heat stress [3], manipulation of crop load and water status to slow ripening [4] and delayed pruning to counter vintage compression [5]. Wine regions around the world are also being challenged by wildfires (or bushfires) which are occurring with increased frequency and severity [6]. Vineyard exposure to smoke can taint grapes due to the absorption of smoke-derived volatile compounds, including volatile phenols [7,8,9], which can impart smoky, medicinal, burnt rubber and ashy characters to wine [8,10,11]. In the last 5 years, fires have affected one or more vintages in prominent wine regions in Australia, Canada, Chile, New Zealand, South Africa and the USA [12,13], and revenue losses arising from ‘smoke taint’ are thought to be in the hundreds of millions of dollars [14,15]. Strategies that mitigate or ameliorate the effects of vineyard smoke exposure are therefore needed.

Early research found that smoke-derived volatile phenols could be removed from wine either by the direct addition of activated carbon [16] or solid phase adsorption following nanofiltration [17], and these methods are still being used by industry to ameliorate smoke-tainted wine. However, ideally, preventative strategies should mitigate smoke taint in the vineyard. To date, few mitigation studies have been performed on grapes or grapevines. Washing grapes (during or after smoke exposure) does not prevent the uptake of smoke-derived volatile phenols by grapes or the perception of smoke attributes in wine [18,19], nor does partial defoliation of grapevines [20]. Several recent studies have evaluated the application of agrochemicals such as kaolin, biofilm, anti-transpirants and activated carbon to grapes or vines as protective sprays [21,22,23,24]. In some instances, promising results were obtained, although the efficacy of the treatment depended on spray coverage [21,22], but some treatments seemed to exacerbate the adsorption of smoke volatiles [22,24]. As such, an effective vineyard-based strategy for preventing smoke taint is yet to be found.

A key challenge associated with field trials evaluating the mitigation of smoke taint is the logistics of achieving reproducible experimental treatments with appropriate controls, both grapevines/grapes which are not exposed to smoke (i.e., negative controls) and grapevines/grapes which are exposed to smoke but without the mitigation treatment(s) (i.e., positive controls). The need for reproducible smoke treatments precludes mitigation trials involving grapevine exposure to wildfire/bushfire smoke, because the occurrence of fires cannot be predicted, the density and duration of smoke exposure is often unknown (and is likely to be highly variable, even within a single vineyard) and there are usually no appropriate controls. Model systems have therefore been developed to overcome these limitations. In the vineyard, purpose-built smoke tents (ranging from ~18 to 60 m^3^) facilitate grapevine exposure to smoke [8,10,18,19,25,26]. This approach enables smoke and mitigation treatments to be applied at different phenological stages during the growing season [25,26], and the intensity of the taint can be influenced according to the duration of smoke exposure [10] and the density of the smoke (i.e., the mass of fuel that is burned) [18]. Several of these studies have attempted to monitor/qualify smoke density using air quality monitors or particulate matter sensors [18,19,25], but the density of smoke achieved in the smoke tents resulted in detector saturation [18,19].

More recently, model systems involving the exposure of excised grape bunches to either smoke (in smoke tents) [23,27] or gaseous volatile phenols (in closed systems, ranging from 6 to 156 L) [24,27] have been used. Surprisingly, the glycosylation of volatile phenols observed in grapes following grapevine exposure to smoke or guaiacol [8,18,19,20,21,28,29] was also found to occur in excised bunches [23,24,27], even in table grapes purchased from retail stores [23,24]. These approaches can therefore be used to generate grapes with elevated concentrations of volatile phenols, in both free and bound (glycosylated) forms. The use of smoke tents and excised bunches provides access to smoke-affected grapes in quantities that allow for winemaking (and therefore chemical and sensory analyses of wine), but smoke treatments need to be applied at or near commercial maturity, because grapes are non-climacteric (i.e., they do not continue to ripen post-harvest). Excised bunches can instead be exposed to different concentrations and/or combinations of gaseous volatile phenols at different phenological stages to simulate smoke exposure (e.g., to study the kinetics of absorption), but the scale of this approach is less suitable for winemaking and sensory analysis (because a 156 L glass tank can only accommodate so many excised bunches). 

Collectively, the model systems described above have enabled researchers to undertake controlled and replicated smoke taint experiments with fewer logistical challenges such as seasonal or environmental constraints and/or restrictions related to safety, the occurrence of fires, and vineyard access [23]. Researchers can simulate smoke exposure to screen prospective mitigation strategies before pursuing more time- and resource-intensive field trials with the most promising strategies. Nevertheless, the extent to which model systems can replicate smoke treatments, and therefore mitigation trials, needs to be validated. This study describes the evaluation of a ‘smoke box’ designed specifically as a model system for exposing grapes to smoke with improved efficiency, flexibility and convenience (relative to field trials involving the use of smoke tents). Importantly, the study sought to evaluate how reproducibly excised bunches could be tainted by smoke, not only between replicate smoke treatments but also within smoke treatments (i.e., taking the potential for spatial variation in the density of smoke into account). The smoke box was subsequently used to compare the efficacy of several agrochemical sprays and protective coverings as methods for mitigating the uptake of smoke-derived volatile phenols by grapes, i.e., the risk of smoke taint. 

## 2. Results and Discussion

### 2.1. Evaluation of the Purpose-Built Smoke Box (Repeatability Trial)

**Figure 1:**
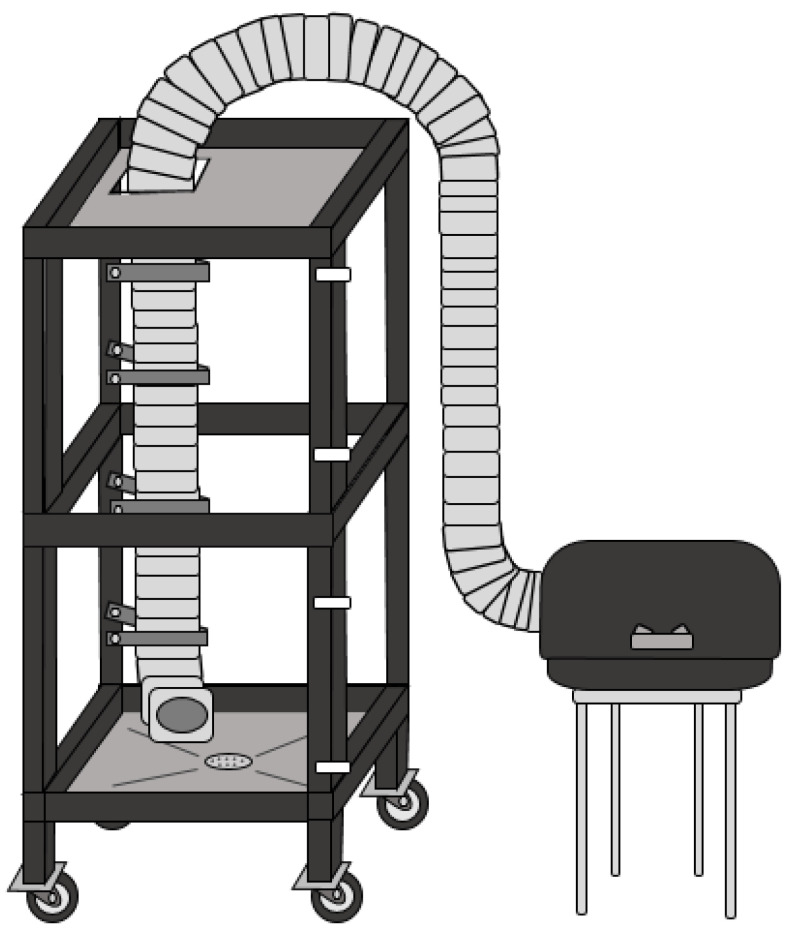
Schematic of the purpose-built smoke box.

To evaluate the reproducibility of smoke treatments (both the spatial variation in smoke density during individual treatments and the variation among three replicate smoke treatments), mature bunches of Semillon grapes were suspended in the smoke box depicted in Figure 1 (and described in more detail in Section 3.1) in a 3 × 3 array (i.e., evenly spaced both horizontally (left, centre and right) and vertically (top, middle and bottom)) and exposed to smoke. Preliminary experiments confirmed that the smoke density in the box depended on the mass of fuel burned and the duration of smoke exposure (data not shown). Smoke treatments were therefore standardized in the current study by combusting set quantities of fuel (100 g of barley straw) and removing grape bunches from the smoke box after a set time (30 min).

**Figure 2:**
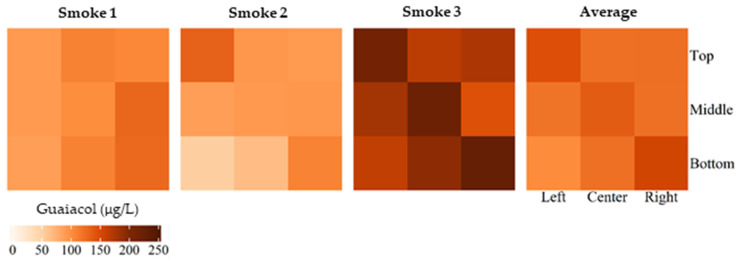
Heat maps depicting spatial variation in the guaiacol concentration of grapes exposed to smoke post-harvest, using the purpose-built smoke box, in replicate smoke treatments and as an average across the three smoke treatments.

Whereas the volatile phenols measured as markers of smoke taint (i.e., guaiacol, 4-methylguaiacol, phenol, cresols, syringol and 4-methylsyringol) were not detected in the control (unsmoked) grapes, they were found at elevated concentrations in grapes following smoke exposure. The heat maps shown in Figure 2 visualize the variation in guaiacol concentration, both by bunch position and by replicate smoke treatment. Bunches positioned in the top left and bottom right of the box tended to have higher guaiacol concentrations, whereas bunches at the bottom left of the box had the lowest guaiacol concentrations. This likely reflects the initial anticlockwise trajectory of smoke as it entered the box, after which the smoke dispersed to fill the box; nevertheless, the ~1 min required to achieve complete obscuration may account for the observed spatial variation in volatile phenol concentrations (Figure 2). Similar results were observed for the other smoke-derived volatile phenols that were measured (Figure S1).

Guaiacol, phenol, *o*-cresol, *m*-cresol and syringol were the most abundant grape volatile phenols (Table 1), at 90–187, 121–220, 38–66, 30–53 and 18–71 µg/L, respectively, in agreement with previous research [18]. When volatile phenol concentrations were compared as means of each replicate smoke treatment (i.e., irrespective of bunch position), statistically significant differences in the composition of smoke-exposed grapes were apparent. Volatile phenols were significantly higher in grapes from the third smoke treatment, while significant differences in phenol, *o*-cresol, syringol and 4-methylsyringol were also observed between the first two smoke treatments (Table 1). This demonstrates the difficulty of exactly replicating smoke treatments. The variation observed in grape volatile phenols reflected the variation in smoke density attributable to a combination of incomplete combustion of fuel and the inevitable loss of some smoke from the smoker (which did not fully seal to give a closed system) and, to a lesser extent, from the fitting connecting the smoker and the exhaust ducting. It was slightly cooler (22–24 °C) during the first two smoke treatments, with a slight breeze that may have contributed to some loss of smoke, whereas during the third treatment, it was slightly warmer (25 °C) but still (i.e., there was no wind). During windy conditions, greater smoke loss might occur, potentially resulting in greater variation in smoke density between smoke replicates. 

When grape volatile phenol concentrations were instead compared according to the position of the excised bunches during the smoke treatment (i.e., as the means of each bunch position across replicate smoke treatments), differences in the composition of smoke-exposed grapes were again apparent (e.g., guaiacol concentrations ranged from 100 to 154 µg/L). However, differences were not statistically significant, due to the variation between replicate smoke treatments (Table S1). This variation has also been encountered with field trials involving the application of smoke to grapevines using smoke tents, and can result in elevated standard deviation/error values for smoke taint marker concentrations [18]. Nevertheless, the use of the smoke box reduces the likelihood that insufficient smoke will be applied to achieve detectable levels of smoke taint. Mitigation trials need to account for the possibility of variation between replicate smoke treatments; i.e., by ensuring experimental treatments are adequately controlled and replicated across smoke treatments. Where mitigation strategies are effective, changes in volatile phenol concentrations will easily exceed any variation observed between replicate smoke treatments, but where the variation is such that the mitigating effect is difficult to ascertain, it likely suggests that the strategy is not capable of providing meaningful protection from smoke exposure. 

Upon completion of the replicate smoke treatments, three excised bunches of Semillon grapes were suspended in the box for 48 h to evaluate the potential for grapes to absorb volatile phenols from the smoke residue that remained. Elevated concentrations of volatile phenols were detected following exposure to smoke residue (Table 1), with guaiacol, phenol, *o*-cresol, *m*-cresol and syringol again being the most abundant at 55, 149, 46, 28 and 15 µg/L, respectively, which were ~40–90% of the average concentrations observed for grapes exposed to smoke in the box (i.e., concentrations averaged across both bunch position and smoke treatment). While residual smoke was not expected to meaningfully contribute to the uptake of volatile phenols by grapes during the 30 min smoke treatments used in the current study (i.e., due to carryover), the results highlight the need for/importance of adequately cleaning/airing the box between experimental trials. 

Previous research has demonstrated the glycosylation of volatile phenols in fruit and/or leaves following grapevine exposure to either smoke or volatile phenols [8,18,19,20,21,28,29], and likely occurs (through the action of glucosyltransferase enzymes) to mitigate the risk of cellular damage [30]. More recent studies have shown that glycosylation also occurs following post-harvest exposure of grapes to smoke or volatile phenols, i.e., in excised bunches [23,24,27], including in table grapes [23,24]. Similar results were obtained in the current study. Control grapes comprised ≤ 7 µg/kg of the volatile phenol glycoconjugates that were measured, but substantial quantities of several glycoconjugates accumulated in the week after the grapes were exposed to smoke in the box (Table 2); in particular, the pentose-glucosides of guaiacol, phenol and cresols were quantitated (as syringol glucose-glucoside (gentiobioside) equivalents) at 242, 216 and 213 µg/kg, respectively. Again, this was in agreement with the results from previous research [18,21,27]; however, some important differences were observed in the volatile phenol glycoside profiles reported in these studies. 

Table S2 presents a cross-study comparison of volatile phenol glycoconjugate concentrations observed in grapes following exposure to either smoke or gaseous volatile phenols under different experimental conditions. One of these studies monitored the accumulation of volatile phenol glycoconjugates in smoke-affected Cabernet Sauvignon grapes [18] and reported glycoconjugate concentrations (i.e., 89–217 µg/kg) 1 week after smoke exposure that were comparable to those observed in excised Semillon bunches in the current study; the notable exceptions being rutinosides of guaiacol and phenol, which were <30 µg/kg in the Cabernet Sauvignon grapes. These results were also in agreement with an earlier study that monitored glycoconjugate accumulation in smoke-affected Merlot grapes [21] and reported rutinosides of guaiacol and phenol at 22 and 26 µg/kg, respectively, relative to the aforementioned volatile phenol pentose glucosides, which were present at 113–300 µg/kg. In contrast, cresol rutinoside concentrations were surprisingly consistent across these three studies, at 114, 89 and 113 µg/kg for Semillon, Cabernet Sauvignon [18], and Merlot [21] grapes, respectively. This suggests that the enzymes responsible for transforming volatile phenols into rutinosides are not inhibited by bunch excision, although glycosylation might be influenced by substrate substitution patterns. This is an important consideration, given that gentiobiosides and rutinosides represent the key glycoconjugates monitored by some commercial laboratories for screening grapes (and wine) for smoke taint, based on their strong association with smoke taint’s sensory attributes [11]. The abundance of pentose glucosides following smoke exposure of excised bunches may influence the perceived efficacy of mitigation trials and/or the intensity of smoke-related sensory attributes in wines, and warrants further investigation. 

Another notable difference in the glycoconjugate profiles was that of syringol gentiobioside concentrations. In previous studies involving the application of smoke to Cabernet Sauvignon and Merlot grapevines, syringol glucose glucosides (gentiobiosides) were amongst the most abundant glycoconjugates observed in grapes (7 days after smoke exposure and at harvest). However, in the current study, and in studies involving the exposure of excised bunches to gaseous phenols [24,27], the concentrations of both syringol and its glucose glucoside (gentiobioside) were comparatively lower than those observed in grapes harvested from smoke-exposed grapevines [18,21]. It is not clear if this reflects the use of excised bunches or other experimental conditions, e.g., fruit maturity at the time of exposure, grape variety or the relative volatility of different phenols.

Although the application of smoke to grapevines or grape bunches presents inherent logistical challenges, a major benefit compared with the exposure of grape bunches to gaseous volatile phenols [24,27] as an alternate model system is that smoke-exposed grapes can be taken through to a winemaking outcome for sensory analysis; however, in the case of excised bunches, smoke exposure would need to occur at or near maturity. The use of gaseous volatile phenols offers the benefit of regulating the quantity of volatile phenols being applied to grapes, which may afford opportunities to investigate the kinetics of uptake and/or biochemical metabolism of volatile phenols (e.g., to resolve knowledge gaps relating to volatile phenol/glycoconjugate mass balance) [18]. Each of the model systems described above afford different advantages and disadvantages, and the most suitable option will depend on the research aim(s) to be investigated (e.g., Figure S2). The smoke box serves as a compromise between the use of smoke and the convenience inherent to its smaller scale.

### 2.2. Application of the Purpose-Built Smoke Box (Mitigation Trial)

The second aim of this study was to demonstrate the potential for the smoke box to be used to evaluate novel strategies for mitigating the risk of smoke taint in grapes. 

In a preliminary field trial involving the application of smoke to Semillon grapevines (using smoke tents), the extent to which an activated carbon (AC) fabric could protect grapes from exposure to smoke was evaluated. Immediately prior to smoke exposure, a number of grape bunches were individually enclosed in bags made from the AC fabric and, for comparative purposes, adjacent grape bunches were similarly enclosed in plastic and paper bags (Figure S3). Following smoke exposure, grape volatile phenol concentrations were compared in the control, smoke-affected and bagged/smoke-affected bunches. 

Control grapes did not contain detectable levels of volatile phenols, but smoke exposure resulted in grapes with guaiacol, syringol, *o*- and *m*-cresol and 4-methylguaiacol concentrations of 21, 16, 8.7, 7.0 and 4.3 µg/kg, respectively (Table 3). In contrast, grapes that were enclosed in plastic or paper bags contained significantly lower volatile phenol concentrations. Guaiacol levels were approximately 50% lower, while other volatile phenol concentrations were ~48–88% lower. Food packaging (including plastic and paper bags) are known to be permeable to small molecules, including aroma volatiles, to different degrees [31]. It is therefore not surprising that in the current study, smoke-derived volatile phenols were detected in grape bunches enclosed in plastic and paper bags. Previous studies that sought to investigate the uptake and glycosylation of exogenous oak volatile compounds by grapevine leaves and fruit reported similar results [28,32], e.g., the presence of the analytes of interest in grapes that were enclosed in plastic bags (as protective barriers) prior to foliar applications of oak extracts or oak volatiles, due to their permeation through the packaging [31]. The AC fabric seemingly provided superior protection, resulting in grapes that contained just 1.3 µg/L of guaiacol and no other detectable smoke-derived volatile phenols (Table 3). These results suggested that the AC fabric adsorbed the vast majority of volatile smoke compounds, preventing their permeation and thus, contamination of the enclosed grapes. 

The smoke box was subsequently used to further validate the potential of the AC fabric to mitigate the uptake of smoke-derived volatile phenols by grapes, alongside two agrochemical sprays, an anti-transpirant and kaolin (a clay-based barrier coating, typically used to protect grapes from sun damage [21]). Paper bags were again included for comparison, but not plastic bags, given their propensity for condensation, which promotes microbial spoilage. The same experimental conditions used in the repeatability trial (i.e., 100 g of straw as fuel and 30 min exposure of excised bunches to smoke) were again used in the mitigation trial, ensuring dense smoke treatments and thus testing the efficacy of each mitigation strategy. To overcome potential variation in smoke density between treatments (as occurred in the repeatability trial), each mitigation treatment was undertaken in triplicate, both within and between three replicate smoke treatments (i.e., *n* = 9 in total). 

Smoke exposure again resulted in Semillon grape bunches with significantly elevated volatile phenol concentrations: i.e., 231 µg/L of guaiacol, 354 µg/L of phenol, 103 µg/L of *o*-cresol and 78 µg/L of syringol (Table 4). Volatile phenol concentrations were several times higher than those observed in the preliminary field trial (Table 3) because the smoke box enabled applications of smoke that were much denser than achieved in the field using the smoke tent. Some variation was again observed between replicate smoke treatments, with the second replicate resulting in significantly higher grape volatile phenol concentrations than the first and third smoke replicates; for example, guaiacol concentrations were 291 µg/L, compared with 186 and 215 µg/L, respectively (data not shown). However, this variation was accounted for by replicating mitigation treatments across replicate smoke treatments, with treatment replicates (excluding the control) positioned randomly within the box during each smoke replicate.

Of the four mitigation strategies that were evaluated, the AC fabric was by far the most effective: enclosing grape bunches in activated carbon fabric prevented the uptake of up to 98% of the smoke-derived volatile phenols that were observed in the smoke-affected grapes (Table 4). Indeed, the volatile phenol levels in grapes enclosed in AC fabric were only 1–7 µg/L. Activated carbon fabrics are used as adsorbents in various industries [33], and activated carbon is routinely used as a fining agent in the wine industry, including for remediating smoke tainted wine [16]. However, this is the first study to demonstrate the capacity of AC fabric to mitigate the risk of smoke taint. The application of bags to individual grape bunches is neither practical nor financially viable, so further research and development is needed, but these results demonstrate proof-of-concept.

The paper bags again afforded the excised bunches reasonable protection from smoke exposure, and, with the exception of *p*-cresol (which was present at very low levels even in smoke-affected grapes), enclosing bunches in paper bags prevented the uptake of ~68 to 81% of each volatile phenol and, seemingly, 100% of syringol (Table 4). The apparent selectivity of protection from different volatile phenols might reflect the molecular size and/or the paper bag’s porosity and surface chemistry [31]. The interior of the paper bag was coated with a wax layer to inhibit moisture loss, and it is possible that following diffusion through the paper layer, syringol and 4-methylsyringol were retained by the hydrophobic wax, such that they did not permeate into the bag and the grapes within. 

Of the two agrochemicals applied to bunches prior to smoke exposure, neither provided meaningful protection. Significantly lower levels of phenol and *m*-cresol were detected in kaolin pre-treated grapes compared with smoke-affected grapes, while the anti-transpirant treatment typically yielded the highest grape volatile phenol concentrations, suggesting this mitigation strategy may actually have facilitated the uptake of smoke-derived volatile phenols by grapes. This is reasonable, given that the active ingredient in the anti-transpirant is a carboxylated hydrophilic polymer, which may well have affinity for smoke-derived volatile compounds. 

Importantly, these results were in agreement with findings from recent studies that evaluated various agrochemicals as protective sprays for the mitigation of smoke taint [21,22,23,24]. Foliar applications of kaolin prior to smoke exposure achieved an 80% reduction in guaiacol glycoconjugates in Merlot grapes at harvest (relative to the corresponding smoke-affected Merlot grapes), but only 40% reductions were achieved when kaolin was applied to Chardonnay grapes and no significant differences were observed following kaolin applications to Sauvignon Blanc grapes [21]. The same anti-transpirant was evaluated as a smoke taint mitigation treatment in a recently published trial [24], albeit under different experimental conditions. In that study, there was no significant difference in the composition (i.e., free or bound volatile phenol concentrations) of Muscat Gordo or Shiraz grapes exposed to gaseous volatile phenols, with or without prior treatment with the anti-transpirant. However, it was observed that the application of other hydrophobic products (e.g., Biopest^®^ Paraffinic Oil, Victoria Fruit Drying Oil, and Parka Plus) significantly increased the concentration of volatile phenols (and their glycoconjugates) in both varieties, a finding consistent with earlier studies that evaluated the influence of lipid-based fungicides on the uptake of volatile phenols [22,23]. Promising results were initially obtained through the application of a synthetic grape cuticle [22], but the outcome could not be replicated in a subsequent growing season. These results reflect the challenge in achieving effective spray coverage, but also suggest that some viticultural practices, e.g., the use of fungicides to manage disease pressure in cooler, wetter areas or anti-transpirants to mitigate water stress in hotter, drier areas, might exacerbate the risk of smoke taint in the event of a nearby bushfire/wildfire.

In conclusion, the results presented herein demonstrate the potential for the smoke box to be used as a rapid, convenient approach to smoke taint mitigation research, overcoming the logistical constraints associated with vineyard-based field trials, as well as a very promising strategy for preventing smoke taint, i.e., activated carbon fabric.

## 3. Materials and Methods

### 3.1. Purpose-Built Smoke Box

A purpose-built smoke box (0.8 m × 0.8 m × 1.5 m, 0.96 m^3^, Figure 1) comprising a steel frame fitted with glass panes (as walls) and aluminium sheeting (as the ceiling and floor), and sealed with silicone rubber was constructed by the University of Adelaide’s School of Physical Sciences mechanical workshop. One wall was lined with a self-adhesive weather strip and mounted as a door, fitted with four metal latches, while the floor was angled downwards to a centrally positioned drain to facilitate cleaning. The smoke box was also fitted with swivel plate castors to allow it to be easily moved. Flexible aluminium exhaust ducting (125 mm × 3 m) was mounted in the left rear corner of the box, running from the floor and out via the ceiling for connection to a commercial fire box smoker (CharGriller, www.chargrilleraustralia.com.au (accessed on 21 February 2022)). This enabled fuel to be combusted in the smoker and the resulting smoke to be carried into the smoke box.

### 3.2. Field Trial

A preliminary field trial involving the exposure of Semillon grapevines to smoke (for 1 h, approximately 2 days before maturity when TSS was ~21 °Brix) was conducted in a vineyard at the University of Adelaide’s Waite Campus in Urrbrae, South Australia (34°58′ S, 138°38′ E). Three adjacent vines were enclosed in a purpose-built smoke tent (2.0 m × 6.0 m × 2.5 m) and barley straw (~2 kg) combusted portionwise (i.e., over the hour) in two commercial smokers (as described previously [18]) to maintain smoke production. Prior to the smoke treatment, grape bunches (one per vine, per treatment) were enclosed in plastic, paper or activated carbon (AC) felt bags (approximately 25 cm × 20 cm each). The plastic and paper bags were purchased from a supermarket, while the AC felt bags were made in-house from a commercial AC fibre felt (Nature Technologies, Hangzhou, China). The bagged bunches were harvested immediately after smoke exposure, together with the smoke-exposed bunches (one per vine) and control bunches (three from a Semillon vine that had not been exposed to smoke). Grapes were separated from the rachis and homogenized with a T18 Ultra Turrax (IKA, Saufen, Germany). The resulting grape homogenate was frozen at −4 °C until needed for volatile phenol analysis.

### 3.3. Box Trials

#### 3.3.1. Repeatability Trial

A trial involving the exposure of excised bunches of grapes to smoke using the purpose-built smoke box was performed in triplicate to test both the repeatability of the smoke treatments and the extent to which the position of grape bunches within the smoke box influenced their level of taint. Grape bunches (30 in total) were harvested (at maturity, when TSS was ~22–23 °Brix) from (control) Semillon grapevines from the field trial described in Section 3.2. Three replicated smoke treatments were performed, each involving the exposure of 9 grape bunches to smoke (for 30 min), with the bunches positioned in the smoke box in a 3 × 3 array: top left, top centre, top right, middle left, middle centre, middle right, bottom left, bottom centre and bottom right. Barley straw (~100 g per treatment) was combusted in the fire box (as above) and the duration of smoke exposure commenced when smoke was first observed in the box (i.e., exiting the exhaust duct). Grape samples (50 berries per bunch per trial, chosen randomly) were collected immediately after smoke exposure and homogenized (as above), and the resulting grape homogenate was frozen at −4 °C until needed for volatile phenol analysis. After sampling, the 9 bunches remaining from the third replicate box trial were stored in darkness at 21 °C for 1 week. Grape samples (50 berries per bunch, chosen randomly) were again collected, homogenized and frozen at −4 °C until needed for volatile phenol glycoconjugate analysis. Volatile phenol glycoconjugate concentrations were also quantified in the control grape homogenate (i.e., homogenate derived from control grapes from the field trial described in Section 3.2). Upon completion of the smoke treatments, the three remaining grape bunches were suspended in the box for 48 h to evaluate the potential uptake of volatile phenols from residual smoke. Grapes were again sampled and homogenized for chemical analysis, as above. 

#### 3.3.2. Mitigation Trial

A separate trial involving the exposure of excised bunches of grapes to smoke using the purpose-built smoke box was performed (also in triplicate) to evaluate the efficacy of four strategies for mitigating the compositional effects of smoke on grapes: the use of powdered kaolin (a clay-based sunscreen, trade name Surround, sourced from AgNova Technologies; Box Hill, VIC, Australia) and an anti-transpirant (trade name Envy^®^, sourced from AgroBest Nutritional Systems; Nerang, QLD, Australia) as protective sprays, and the use of paper and AC felt bags (as described in Section 3.2) as protective coverings. Grape bunches (45 in total) were again harvested from (control) Semillon grapevines from the field trial described in Section 3.2 (at maturity when TSS was ~22–23 °Brix). Kaolin (prepared as a 50 g/L aqueous solution) and Envy (prepared as a 50 mL/L aqueous solution) were applied liberally to the grape bunches (using hand-held pump-action spray bottles) 24 h prior to harvest and smoke exposure. Three replicated smoke treatments were performed (as described in Section 3.3.1), each involving the exposure of 15 grape bunches (i.e., three replicates per treatment, including a smoke-only treatment) to smoke (for 30 min), with the bunches randomly positioned in the smoke box in a 5 × 3 array, positioned at the same height, in the centre of the box. Grape samples (50 berries per bunch per treatment per replicate, chosen randomly) were again collected immediately after smoke exposure and homogenized (as above), and the resulting grape homogenate was frozen at −4 °C until needed for volatile phenol analysis.

### 3.4. Chemical Analysis

The concentrations of smoke-derived volatile phenols (guaiacol; 4-methylguaiacol; phenol; *o*-, *m*- and *p*-cresol; syringol and 4-methylsyringol) were measured in grape juice or homogenate using an Agilent 6890 gas chromatograph coupled to a 5973 mass selective detector (Agilent Technologies, Forest Hill, VIC, Australia) according to previously published stable isotope dilution analysis (SIDA) methods [29,34], using d_4_-guaiacol (synthesized in-house, as described previously [35]) and d_3_-syringol (CDN Isotopes, Pointe-Claire, QC, Canada) as internal standards. Data acquisition and processing were performed using ChemStation (version B.04.03, Agilent Technologies) and MassHunter software. Field trial samples were analysed by the Australian Wine Research Institute’s (AWRI) Commercial Services Laboratory (Adelaide, SA, Australia). Volatile phenol glycoconjugate concentrations (measured as syringol gentiobioside equivalents) were also measured in grape homogenate using an Agilent 1200 high-performance liquid chromatograph equipped with a 1290 binary pump coupled to an AB SCIEX Triple Quad^TM^ 4500 tandem mass spectrometer, with a Turbo V^TM^ ion source (Framingham, MA, USA), and previously published SIDA methods [29]; d_3_-syringol gentiobioside (Toronto Research Chemicals, Toronto, ON, Canada) was used as the internal standard. Data acquisition and processing were performed using Analyst software (version 1.7 AB SCIEX). The limits of quantitation for volatile phenols and volatile phenol glycoconjugates were 1–2 and 1 µg/L, respectively.

### 3.5. Data Analysis and Visualization

Compositional data were analysed by one-way analysis of variance using R statistical software (version 4.0.3, Cambridge, MA, USA), with mean comparisons performed by Tukey’s honest significant difference test at a significance level of α < 0.05. Heatmaps were generated using the “Complex Heatmap” package in R. 

## Figures and Tables

**Table 1:** Concentration of volatile phenols (µg/L) in juice from control grapes and grapes exposed to smoke (or smoke residue).

Treatment	Guaiacol	4-Methyl Guaiacol	Phenol	*o*-Cresol	*m*-Cresol	*p*-Cresol	Syringol	4-Methyl Syringol
Control	nd	nd	na	nd	nd	nd	nd	nd
Smoke 1	105 ± 5 b	17 ± 0.8 b	121 ± 7 c	38 ± 2 c	30 ± 2 b	6 ± 0.5 b	50 ± 2 b	7 ± 0.2 b
Smoke 2	90 ± 8 bc	14 ± 1.4 b	176 ± 15 ab	52 ± 5 b	36 ± 3 b	8 ± 1 b	18 ± 2 c	2 ± 0.1 c
Smoke 3	187 ± 9 a	31 ± 1.6 a	220 ± 10 a	66 ± 3 a	53 ± 3 a	13 ± 1 a	71 ± 3 a	9 ± 0.3 a
Smoke Residue	55 ± 2 c	7 ± 0.7 c	149 ± 18 bc	46 ± 1 bc	28 ± 2 b	8 ± 3 b	15 ± 2 c	2 ± 0.1 c
*p*	<0.001	<0.001	<0.001	<0.001	<0.001	<0.001	<0.001	<0.001

Values are means of three replicates (*n* = 3) ± standard error for control and residual smoke samples or and nine replicates (*n* = 9) ± standard error for replicate smoke samples. nd = not detected; na = not available. Different letters (within columns) indicate statistical significance (*p* = 0.05, one-way ANOVA).

**Table 2:** Concentration of volatile phenol glycoconjugates (µg/kg) in control grapes and grapes exposed to smoke post-harvest, using the purpose-built smoke box, analysed 7 days after smoke exposure.

Treatment	GuPG	GuR	4MGPG	4MGR	PhPG	PhR	CrPG	CrR	SyrGG	4MSGG
Control	2.4 ± 0.1 b	nd	nd	nd	3.7 ± 0.1 b	nd	7.1 ± 0.5 b	1.4 ± 0.1 b	1.0 ± 0.1 b	nd
Smoke	242 ± 27 a	110 ± 10	22 ± 3	27 ± 3	216 ± 31 a	89 ± 11	213 ± 25 a	114 ± 10 a	55 ± 7 a	9 ± 1
*p*	<0.001	-	-	-	0.003	-	<0.001	<0.001	0.001	-

Values are means of three replicates (*n* = 3) ± standard error, measured as syringol glucose-glucoside equivalents, for control grapes or nine replicates (*n* = 9) ± standard error for smoke-affected grapes. nd = not detected. Different letters (within columns) indicate statistical significance (*p* = 0.05, one-way ANOVA). Gu, guaiacol; 4MG, 4-methylguaiacol; Ph, phenol; Cr, cresol; Syr, syringol; 4MS, 4-methylsyringol; PG, pentose-glucoside; GG, glucose-glucoside; R, rutinoside.

**Table 3:** Concentrations of volatile phenols (µg/kg) in control grapes, smoke-exposed grapes, and grapes enclosed in paper, plastic or activated carbon fabric bags (as protective coverings) during grapevine exposure to smoke.

Treatment	Guaiacol	4-Methyl Guaiacol	*o*-Cresol	*m*-Cresol	*p*-Cresol	Syringol	4-Methyl Syringol
Control	nd	nd	nd	nd	nd	nd	nd
Smoke	21 ± 2.9 a	4.3 ± 0.9 a	8.7 ± 0.9 a	7.0 ± 1.2 a	nd	16 ± 1.7 a	nd
Paper Bag + Smoke	10 ± 1.0 b	1.5 ± 0.5 b	4.5 ± 0.5 b	3.0 ± 0.1 b	nd	2.0 ± 0.1 b	nd
Plastic Bag + Smoke	11 ± 3.3 b	1.7 ± 0.9 b	3.0 ± 1.0 b	2.0 ± 0.6 b	nd	5.3 ± 1.5 b	nd
AC Fabric Bag + Smoke	1.3 ± 0.7 c	nd	nd	nd	nd	nd	nd
*p*	<0.001	0.004	<0.001	<0.001	-	<0.001	-

Values are means of three replicates (*n* = 3) ± standard error. nd = not detected. Different letters (within columns) indicate statistical significance (*p* = 0.05, one-way ANOVA).

**Table 4:** Concentrations of volatile phenols (µg/L) in juice from control grapes, smoke-exposed grapes, and grapes treated with anti-transpirant or kaolin (as protective sprays) or enclosed in paper or activated carbon (AC) fabric bags (as protective coverings) during smoke exposure.

Treatment	Guaiacol	4-Methyl Guaiacol	Phenol	*o*-Cresol	*m*-Cresol	*p*-Cresol	Syringol	4-Methyl Syringol
Control	nd	nd	na	nd	nd	nd	nd	nd
Smoke	231 ± 16 ab	39 ± 3 ab	354 ± 15 a	103 ± 5 ab	81 ± 4 a	10 ± 1 a	78 ± 10 ab	7.3 ± 1 ab
Anti-transpirant	239 ± 24 a	42 ± 4 a	406 ± 26 a	119 ± 9 a	92 ± 7 a	13 ± 3 a	88 ± 13 a	9.0 ± 2 a
Kaolin	183 ± 19 b	29 ± 4 b	286 ± 27 b	81 ± 8 b	64 ± 7 b	13 ± 0.8 a	58 ± 9 b	5.6 ± 1 b
Paper Bag + Smoke	75 ± 9 c	10 ± 1 c	81 ± 9 c	29 ± 3 c	15 ± 2 c	3.9 ± 0.1 b	nd	1.5 ± 0.0 c
AC Fabric Bag + Smoke	4.5 ± 1 d	2.0 ± 0.1 d	7 ± 2 d	2 ± 0.4 d	2 ± 0.3 c	nd	nd	1.4 ± 0.0 c
*p*	<0.001	<0.001	<0.001	<0.001	<0.001	<0.001	<0.001	<0.001

Values are means of three replicates (*n* = 3). nd, not detected; na, not available. Different letters (within columns) indicate statistical significance (*p* = 0.05, one-way ANOVA).

## Data Availability

All data are included in the article and/or Supplementary Materials.

## Supplementary Materials

The following supporting information can be downloaded online. Figure S1: Heat maps depicting spatial variation in the (a) phenol, (b) *o*-cresol, (c) *m*-cresol, and (d) syringol concentrations of grapes exposed to smoke post-harvest using the purpose-built smoke box, by replicate smoke treatments and as an average across the three smoke treatments. Figure S2: Photograph showing Semillon grape bunches enclosed in plastic, paper and activated carbon fabric bags prior to treatments involving grapevine exposure to smoke. Figure S3: Comparison of natural, experimental and model smoke exposure as tools to pursue different smoke taint research aims, and their relative advantages and limitations. Table S1: Concentrations of volatile phenols (µg/L) in grapes exposed to smoke post-harvest, according to the spatial position of bunches within the smoke box. Table S2: Cross-study comparison of volatile phenol glycoconjugate concentrations (µg/kg) measured in grapes following exposure to smoke or gaseous volatile phenols, under different experimental conditions.

## Sample Availability

Samples of compounds are not available from the authors.

## References

[1] Van Leeuwen C., Darriet P. (2016). The impact of climate change on viticulture and wine quality. J. Wine Econ..

[2] Serra I., Strever A., Myburgh P.A., Deloire A. (2014). Review: The interaction between rootstocks and cultivars (*Vitis vinifera* L.) to enhance drought tolerance in grapevine. Aust. J. Grape Wine Res..

[3] Caravia L., Pagay V., Collins C., Tyerman S.D. (2017). Application of sprinkler cooling within the bunch zone during ripening of Cabernet Sauvignon berries to reduce the impact of high temperature. Aust. J. Grape Wine Res..

[4] Previtali P., Dokoozlian N.K., Pan B.S., Wilkinson K.L., Ford C.M. (2021). Crop load and plant water status influence the ripening rate and aroma development in berries of grapevine (*Vitis vinifera* L.) cv. Cabernet Sauvignon. J. Agric. Food Chem..

[5] Moran M.A., Bastian S.E., Petrie P.R., Sadras V.O. (2021). Impact of late pruning and elevated ambient temperature on Shiraz wine chemical and sensory attributes. Aust. J. Grape Wine Res..

[6] Bowman D.M.J.S., Kolden C.A., Abatzoglou J.T., Johnston F.H., van der Werf G.R., Flannigan M. (2020). Vegetation fires in the Anthropocene. Nat. Rev. Earth Environ..

[7] Krstic M.P., Johnson D.L., Herderich M.J. (2015). Review of smoke taint in wine: Smoke-derived volatile phenols and their glycosidic metabolites in grapes and vines as biomarkers for smoke exposure and their role in the sensory perception of smoke taint. Aust. J. Grape Wine Res..

[8] Ristic R., Fudge A.L., Pinchbeck K.A., De Bei R., Fuentes S., Hayasaka Y., Tyerman S.D., Wilkinson K.L. (2016). Impact of grapevine exposure to smoke on vine physiology and the composition and sensory properties of wine. Theor. Exp. Plant. Physiol..

[9] Noestheden M., Thiessen K., Dennis E.G., Zandberg W.F. (2017). Quantitating organoleptic volatile phenols in smoke-exposed *Vitis vinifera* berries. J. Agric. Food Chem..

[10] Kennison K.R., Gibberd M.R., Pollnitz A.P., Wilkinson K.L. (2008). Smoke-derived taint in wine: The release of smoke-derived volatile phenols during fermentation of Merlot juice following grapevine exposure to smoke. J. Agric. Food Chem..

[11] Parker M., Osidacz P., Baldock G.A., Hayasaka Y., Black C.A., Pardon K.H., Jeffery D.W., Geue J.P., Herderich M.J., Francis I.L. (2012). Contribution of several volatile phenols and their glycoconjugates to smoke-related sensory properties of red wine. J. Agric. Food Chem..

[12] Mirabelli-Montan Y.A., Marangon M., Graça A., Mayr Marangon C.M., Wilkinson K.L. (2021). Techniques for mitigating the effects of smoke taint while maintaining quality in wine production: A review. Molecules.

[13] Summerson V., Gonzalez Viejo C., Pang A., Torrico D.D., Fuentes S. (2021). Review of the effects of grapevine smoke exposure and technologies to assess smoke contamination and taint in grapes and wine. Beverages.

[14] Claughton D., Jeffery C., Pritchard M., Hough C., Wheaton C. Wine Industry’s ‘Black Summer’ as Cost of Smoke Taint, Burnt Vineyards, and Lost Sales Add Up. ABC Rural. https://www.abc.net.au/news/rural/2020-02-28/fire-and-smoke-costs-wine-industry-40-million-dollars/11972450?utm_campaign=abc_news_web&utm_content=link&utm_medium=content_shared&utm_source=abc_news_web.

[15] Romano A. The Impact of 2020’s Wildfires. Wine Spectator. https://www.winespectator.com/articles/the-impact-of-2020-s-wildfires-063021.

[16] Fudge A.L., Schiettecatte M., Ristic R., Hayasaka Y., Wilkinson K.L. (2012). Amelioration of smoke taint in wine by treatment with commercial fining agents. Aust. J. Grape Wine Res..

[17] Fudge A.L., Ristic R., Wollan D., Wilkinson K.L. (2011). Amelioration of smoke taint in wine by reverse osmosis and solid phase adsorption. Aust. J. Grape Wine Res..

[18] Szeto C., Ristic R., Capone D., Puglisi C., Pagay V., Culbert J., Jiang W., Herderich M., Tuke J., Wilkinson K. (2020). Uptake and glycosylation of smoke-derived volatile phenols by Cabernet Sauvignon grapes and their subsequent fate during winemaking. Molecules.

[19] Noestheden M., Dennis E.G., Zandberg W. (2018). Quantitating volatile phenols in Cabernet Franc berries and wine after on-vine exposure to smoke from a simulated forest fire. J. Agric. Food Chem..

[20] Ristic R., Pinchbeck K.A., Fudge A.L., Hayasaka Y., Wilkinson K.L. (2013). Effect of leaf removal and grapevine smoke exposure on colour, chemical composition and sensory properties of Chardonnay wines. Aust. J. Grape Wine Res..

[21] van der Hulst L., Munguia P., Culbert J.A., Ford C.M., Burton R.A., Wilkinson K.L. (2019). Accumulation of volatile phenol glycoconjugates in grapes following grapevine exposure to smoke and potential mitigation of smoke taint by foliar application of kaolin. Planta.

[22] Favell J.W., Noestheden M., Lyon S.M., Zandberg W.F. (2019). Development and evaluation of a vineyard-based strategy to mitigate smoke-taint in wine grapes. J. Agric. Food Chem..

[23] Favell J.W., Fordwour O.B., Morgan S.C., Zigg I., Zandberg W. (2021). Large-scale reassessment of in-vineyard smoke-taint grapevine protection strategies and the development of predictive off-vine models. Molecules.

[24] Culbert J.A., Krstic M.P., Herderich M.J. (2021). Development and utilization of a model system to evaluate the potential of surface coatings for protecting grapes from volatile phenols implicated in smoke taint. Molecules.

[25] Kennison K.R., Wilkinson K.L., Pollnitz A.P., Williams H.G., Gibberd M.R. (2009). Effect of timing and duration of grapevine exposure to smoke on the composition and sensory properties of wine. Aust. J. Grape Wine Res..

[26] Kennison K.R., Wilkinson K.L., Pollnitz A.P., Williams H.G., Gibberd M.R. (2011). Effect of smoke application to field-grown Merlot grapevines at key phenological growth stages on wine sensory and chemical properties. Aust. J. Grape Wine Res..

[27] Culbert J.A., Jiang W., Ristic R., Puglisi C.J., Nixon E.C., Shi H., Wilkinson K.L. (2021). Glycosylation of volatile phenols in grapes following pre-harvest (on-vine) vs. post-harvest (off-vine) exposure to smoke. Molecules.

[28] Pardo-Garcia A.I., Wilkinson K.L., Culbert J.A., Lloyd N.D.R., Alonso G.L., Salinas M.R. (2017). Accumulation of guaiacol glycoconjugates in fruit, leaves and shoots of *Vitis vinifera* cv. Monastrell following foliar applications of guaiacol or oak extract to grapevines. Food Chem..

[29] Hayasaka Y., Parker M., Baldock G.A., Pardon K.H., Black C.A., Jeffery D.W., Herderich M.J. (2013). Assessing the impact of smoke exposure in grapes: Development and validation of an HPLC-MS/MS method for the quantitative analysis of smoke-derived phenolic glycosides in grapes and wine. J. Agric. Food Chem..

[30] Song C., Härtl K., McGraphery K., Hoffman T., Schwab W. (2018). Attractive but toxic: Emerging roles of glycosidically bound volatiles and glycosyltrasnferases involved in their formation. Mol. Plant..

[31] Siracusa V. (2012). Food packaging permeability behaviour: A report. Int. J. Polym. Sci..

[32] Pardo-Garcia A.I., Wilkinson K.L., Culbert J.A., Lloyd N.D.R., Alonso G.L., Salinas M.R. (2015). Accumulation of glycoconjugates of 3-methyl-4-hydroxyoctanoic acid in fruits, leaves, and shoots of *Vitis vinifera* cv. Monastrell following foliar applications of oak extract or oak lactone. J. Agric. Food Chem..

[33] Chen J.Y. (2017). Activated Carbon Fiber and Textiles.

[34] Pollnitz A.P., Pardon K.H., Sykes M., Sefton M.A. (2004). The effects of sample preparation and gas chromatograph injection techniques on the accuracy of measuring guaiacol, 4-methylguaiacol and other volatile oak compounds in oak extracts by stable isotope dilution analyses. J. Agric. Food Chem..

[35] Crump A.M., Sefton M.A., Wilkinson K.L. (2014). Microwave-assisted deuterium exchange: The convenient preparation of isotopically labelled analogues for stable isotope dilution analysis of volatile wine phenols. Food Chem..

