# Supplementary material for: Thinking Inside the Box: A Novel Approach to Smoke Taint Mitigation Trials
Source: Molecules. 2022 Mar 3;27(5):1667. doi: 10.3390/molecules27051667 (PMC8911903; doi:10.3390/molecules27051667)
Supplement: Supplementary file 1 [file molecules-27-01667-s001.zip › molecules-1603969-supplementary.pdf]

Supplementary Materials

Thinking Inside the Box: a Novel Approach to Smoke Taint Mitigation Trials

Colleen Szeto [1,2], Renata Ristic [1], and Kerry Wilkinson [1,2,*]

[1] Department of Wine Science, Waite Research Institute, The University of Adelaide, PMB 1, Glen Osmond, SA 5064, Australia; colleen.szeto@adelaide.edu.au (C.S.); renata.ristic@adelaide.edu.au (R.R.)

[2] The Australian Research Council Training Centre for Innovative Wine Production, PMB1, Glen Osmond, SA 5064, Australia

* Correspondence: kerry.wilkinson@adelaide.edu.au (K.W.); Tel: +61-8-8313-7360

Table S1. Concentrations of volatile phenols (µg/L) in grapes exposed to smoke post-harvest, according to spatial position of bunches within the smoke box.

Vertical	Horizontal	Guaiacol	4-Methyl Guaiacol	Phenol	*o*-Cresol	*m*-Cresol	*p*-Cresol	Syringol	4-Methyl Syringol
Top	Left	145 ± 36	25 ± 7	199 ± 48	61 ± 15	46 ± 11	12 ± 3	51 ± 13	6 ± 2
Top	Center	122 ± 22	20 ± 4	170 ± 19	49 ± 5	39 ± 4	11 ± 1	47 ± 15	6 ± 2
Top	Right	123 ± 22	22 ± 4	173 ± 20	54 ± 6	40 ± 5	9 ± 2	45 ± 10	6 ± 1
Middle	Left	119 ± 30	20 ± 6	156 ± 28	49 ± 10	37 ± 7	8 ± 3	45 ± 16	6 ± 2
Middle	Center	137 ± 42	22 ± 7	183 ± 43	56 ± 12	43 ± 10	8 ± 2	49 ± 17	6 ± 2
Middle	Right	122 ± 15	20 ± 3	178 ± 13	52 ± 2	39 ± 2	9 ± 3	45 ± 14	6 ± 2
Bottom	Left	100 ± 33	17 ± 6	130 ± 30	39 ± 9	30 ± 8	8 ± 2	41 ± 17	6 ± 2
Bottom	Center	122 ± 37	20 ± 6	152 ± 30	47 ± 9	36 ± 8	6 ± 1	45 ± 17	6 ± 2
Bottom	Right	154 ± 37	25 ± 6	218 ± 37	64 ± 10	50 ± 8	11 ± 4	55 ± 17	7 ± 2
p		0.976	0.981	0.724	0.750	0.773	0.801	0.999	1.000

Values are means of three replicates (n = 3) ± standard error. No statistical significance was detected as a function of bunch position during smoke treatment (p = 0.05, one way ANOVA).

Table S2. Cross-study comparison of volatile phenol glycoconjugate concentrations (μg/kg) measured in grapes following exposure to smoke or gaseous volatile phenols, under different experimental conditions.

Variety	Model System	Timing and Duration of Smoke Exposure	Sampling Time	Sample Density	GuR	CrR	SyGG	MSyGB	CrPG	GuPG	References
Semillon	smoke box	0.5 h, post-harvest	7 d post-smoke	9 bunches/0.96 m^3	110 ± 10	114 ± 10	55 ± 7	9 ± 1	213 ± 25	242 ± 27	current study
Viognier	smoke tent	1 h, 1 week pre-harvest	7 d post-smoke	3 vines/30 m^3	tr	3 ± 0.3	39 ± 9	8 ± 2	21 ± 3	40 ± 2	[27]
Viognier	smoke tent	1 h, post-harvest	7 d post-smoke	3 vines/30 m^3	tr	3 ± 0.3	40 ± 8	9 ± 1	23 ± 0.5	43 ± 2	[27]
Cabernet Sauvignon	smoke tent	1 h, 1 week pre-harvest	7 d post-smoke	3 vines/30 m^3	tr	3 ± 0.5	12 ± 4	2 ± 0.5	3 ± 0.4	3 ± 0.4	[27]
Cabernet Sauvignon	smoke tent	1 h, post-harvest	7 d post-smoke	3 vines/30 m^3	tr	3 ± 0.5	9 ± 2	1 ± 0.3	3 ± 0.2	3 ± 0.3	[27]
Viognier	gaseous phenols	60 h, post-harvest	immediate	3 bunches/0.16 m^3	25 ± 4	205 ± 36	45 ± 5	17 ± 3	2114 ± 135	1111 ± 104	[27]
Cabernet Sauvignon	gaseous phenols	60 h, post-harvest	immediate	3 bunches/0.16 m^3	90 ± 14	571 ± 74	102 ± 17	26 ± 5	1196 ± 223	482 ± 122	[27]
Muscat Gordo[1]	gaseous phenols	60 h, post-harvest	immediate	3 bunches/0.16 m^3	10 ± 4	67 ± 23	88 ± 37	-	299 ± 91	197 ± 54	[24]
Shiraz	gaseous phenols	60 h, post-harvest	immediate	3 bunches/0.16 m^3	24 ± 6	218 ± 51	6 ± 0.5	-	197 ± 19	105 ± 6	[24]
Cabernet Sauvignon	smoke tent	1 h, 7-10 days post-veraison	7 d post-smoke	3 vines/30 m^3	11 ± 2	89 ± 12	455 ± 86	62 ± 10	217 ± 34	185 ± 33	[18]
Merlot	smoke tent	1 h, 14 days post-veraison	7 d post-smoke	3 vines/30 m^3	22 ± 12	113 ± 64	176 ± 96	-	300 ± 156	283 ± 189	[21]

Data are reported as means ± standard error values; tr = trace (i.e., 0.5–1 μg/kg).
Gu = guaiacol; Cr = cresol; Sy = syringol; MSy = 4-methylsyringol; PG = pentose-glucoside; GG = glucose-glucoside (gentiobioside); R = rutinoside.

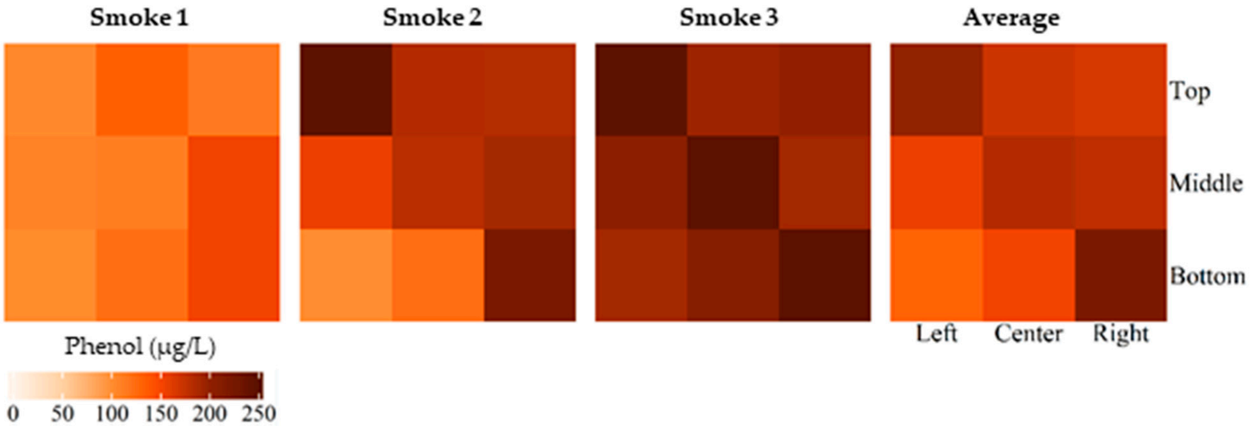

(a)

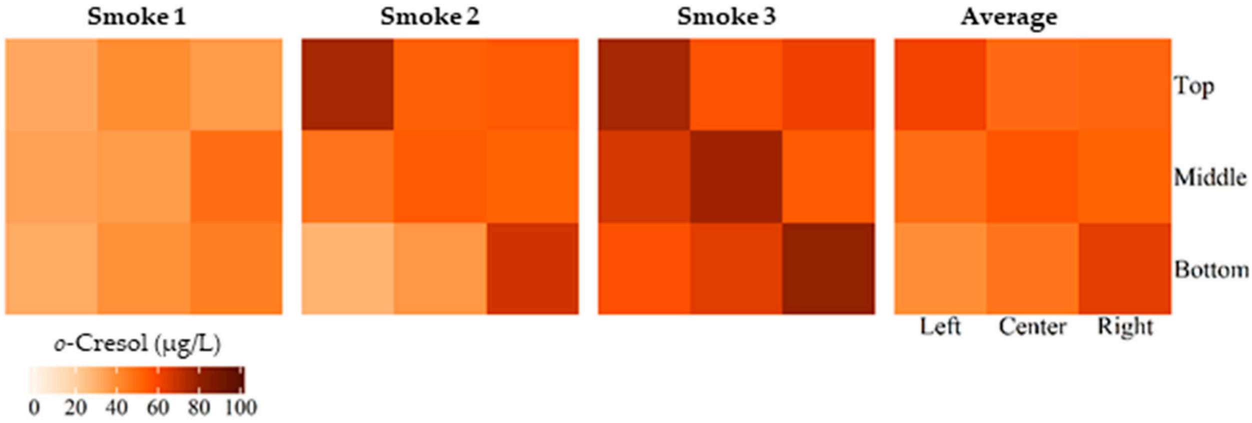

(b)

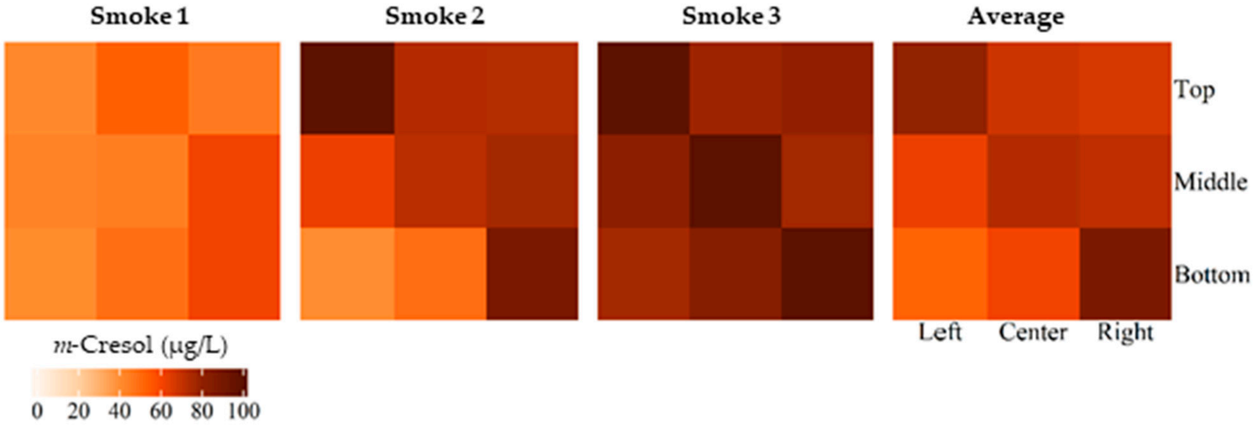

(c)

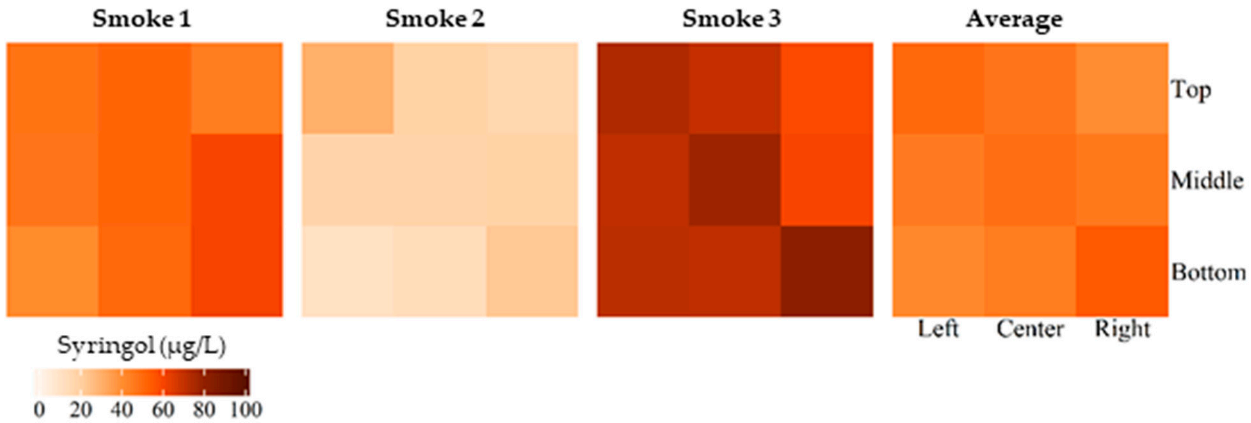

(d)

Figure S1. Heat maps depicting spatial variation in (**a**) phenol, (**b**) *o*-cresol, (**c**) *m*-cresol, and (**d**) syringol concentrations of grapes exposed to smoke post-harvest, using the purpose-built smoke box, by replicate smoke treatments and as an average across the three smoke treatments.

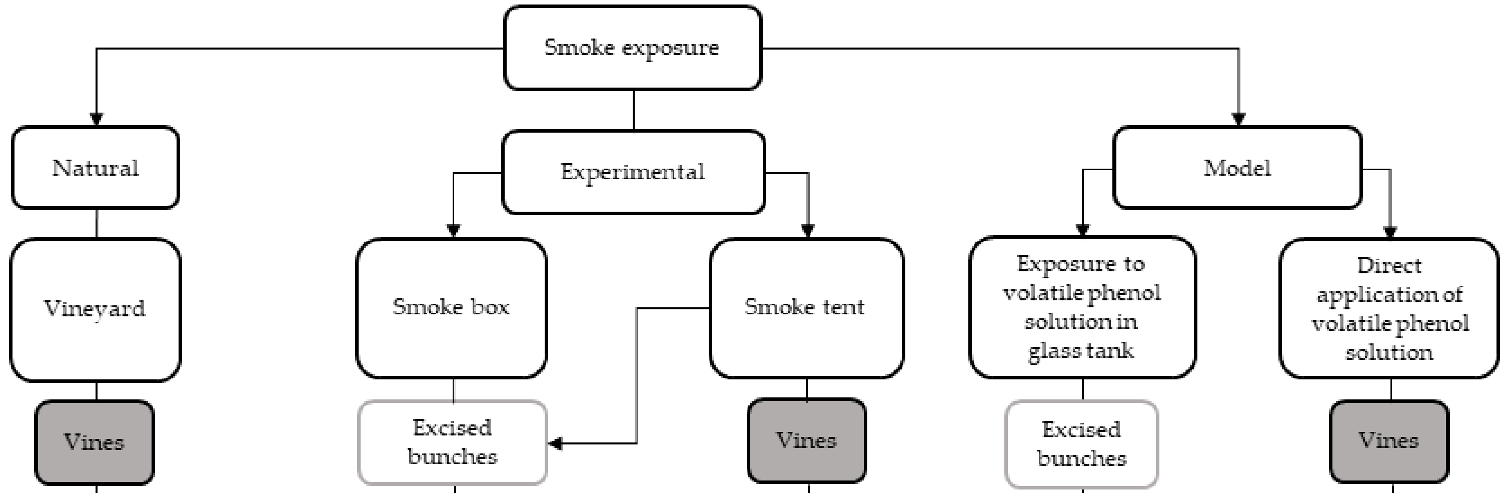

**Resources**	Low	Low-Medium	High	Minimal
**Flexibility**	Low	Medium-High	Medium	High
**Advantages**	Provides unique opportunities to observe impacts of actual bushfire smoke on grape/wine chemical and wine sensory assessments and practical, industry-oriented data interpretation	Provides benefit of actual smoke exposure to a high volume of fruit in less time, enables pursuit of preliminary trials prior to scale-up in the vineyard	Provides most accurate model of actual bushfire smoke, enables pursuit of most diverse research aims, e.g. impacts of grape maturity, vineyard mitigation, and timing of smoke exposure	Provides least accurate model of actual bushfire smoke, but enables pursuit of unique basic research aims, e.g. volatile phenol translocation between leaves and fruit and exposure of vines/bunches to known quantities of volatile phenols
**Limitations**	Limited by vineyard access, uncertainty of bushfire occurrence, and inconsistent/unknown levels of grapevine smoke exposure	Limited by bunch stability post-excision, variable bunch maturity/size/density, and weather	Limited by fruit volume, time-sensitive experimental window, time-intensive experimental Smokes, and weather	Limited by variable bunch size/density, simplification of smoke to a few volatile phenols, and minimal industry relevance

Figure S2. Comparison of natural, experimental and model smoke exposure as tools to pursue different smoke taint research aims, and their relative advantages and limitations.

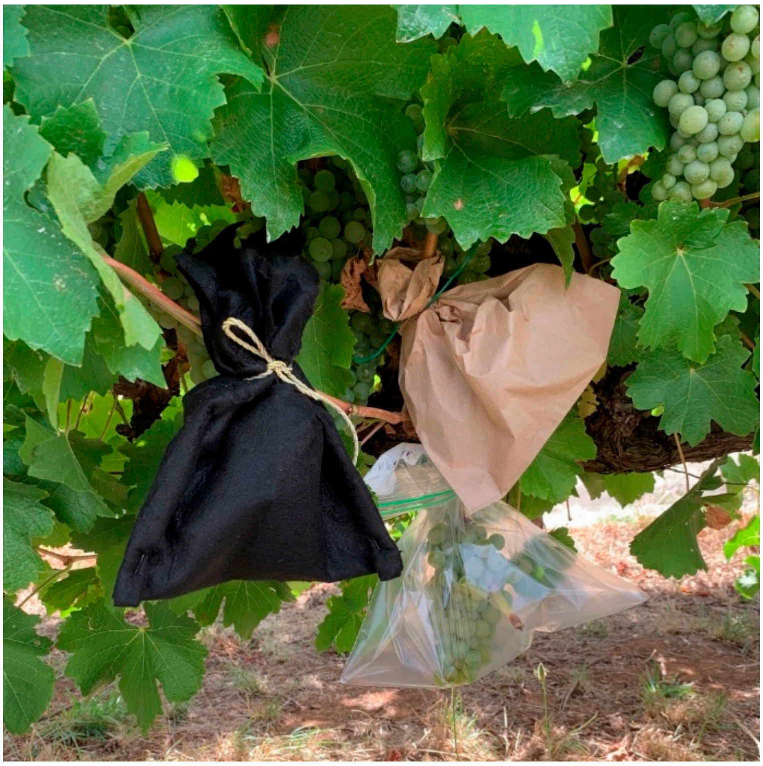

Figure S3. Photograph showing Semillon grape bunches enclosed in plastic, paper and activated carbon fabric bags prior to treatments involving grapevine exposure to smoke.
